# TGF-β signaling promotes tumor vasculature by enhancing the pericyte-endothelium association

**DOI:** 10.1186/s12885-018-4587-z

**Published:** 2018-06-19

**Authors:** Justin Zonneville, Alfiya Safina, Alexander M. Truskinovsky, Carlos L. Arteaga, Andrei V. Bakin

**Affiliations:** 1 Department of Cancer Genetics, Roswell Park Cancer Institute, Buffalo, New York, 14263 USA; 2 Department of Cell Stress Biology, Roswell Park Cancer Institute, Buffalo, New York, USA; 3 Department of Pathology, Roswell Park Cancer Institute, Buffalo, New York, USA; 4 Harold C. Simmons Comprehensive Cancer Center, UT Southwestern Medical Center, Dallas, TX USA

**Keywords:** Tumor microenvironment, Breast cancer, Tumor angiogenesis, Tumor-associated fibroblasts, TGF-beta, Fibronectin, Pericytes

## Abstract

**Background:**

The breast cancer microenvironment promotes tumor vascularization through the complex interactions involving tumor-associated fibroblasts (TAFs). Emerging data indicate that TAFs increase production and signaling by TGF-β cytokines, while the role of TGF-β signaling in the regulation of tumor blood vessels is not fully understood. The current study presents evidence that TAFs enhance the organization of tumor blood capillaries, and TGF-β signaling plays an important role in this response.

**Methods:**

Tumor vascularization was studied in xenograft models of breast carcinoma cells, alone and in combination with fibroblasts. TGF-β signaling in breast cancer cells was modulated by expression of kinase-inactive TGFBR1-K232R (dnTGFBR1) or constitutive-active TGFBR1-T204D (caTGFBR1) receptor mutants. The architecture of tumor blood capillaries was assessed by immune-histochemical analysis of endothelium and pericytes. The role of TGF-β-Smad signaling in fibronectin expression was examined using adenoviral transduction of signaling components.

**Results:**

Our studies revealed that TAFs significantly increase the lumen size of blood microvessels. Inactivation of TGF-β signaling in tumor cells by dnTGFBR1 reduced the microvessel density and lumen sizes, decreasing tumor growth. In contrast, caTGFBR1-tumors exhibited greater vessel density and lumen sizes. Tumors with inactive dnTGFBR1 showed lower amounts of TAFs, while caTGFBR1 increased amounts of TAFs compared to the control. Inspection of pericytes and endothelial cells in tumor vasculature revealed that TAFs enhanced vessel coverage by pericytes, vascular cells supporting capillaries. This effect was impaired in dnTGFBR1-tumors, whereas active caTGFBR1 enhanced the association of pericytes with endothelium. Accordingly, dnTGFBR1-tumors exhibited the presence of hemorrhages, a sign of fragile blood vessels. Biochemical analysis showed that TGFBR1-SMAD signaling up-regulates fibronectin, a prominent regulator of endothelium-pericyte interactions.

**Conclusions:**

The current study indicates that tumor-fibroblast crosstalk enhances tumor vascularization by increasing the pericyte-endothelium association via a mechanism involving the TGFβ-fibronectin axis. The tumor-fibroblast model represents a useful system for dissecting the complex interactions governing tumor angiogenesis and developing new approaches to therapeutic targeting tumor vasculature.

**Electronic supplementary material:**

The online version of this article (10.1186/s12885-018-4587-z) contains supplementary material, which is available to authorized users.

## Background

Breast cancer is the second leading cause of cancer-related death in women [1]. Compelling evidence points to the tumor microenvironment (TME) as a major factor influencing breast cancer progression and response to therapy [2]. In the breast cancer TME, cancer and host stromal cells are engaged in complex and dynamic interactions that promote tumor angiogenesis and obstruct the anticancer immune response. A better understanding of these mechanisms and interactive networks is integral for developing more effective therapies.

Tumor-associated fibroblasts (TAFs) are the predominant cell type in the TME of the most aggressive and difficult to treat cancers [3]. TAFs have been implicated in recruitment of pro-angiogenic myeloid immune cells such as macrophages and myeloid-derived suppressor cells (MDSCs), thereby promoting tumor angiogenesis and metastasis [4, 5]. Pro-angiogenic activity of myeloid cells depends on metalloproteinase MMP9/gelatinase-B [5–7], which releases VEGFA from matrix-deposited sources increasing recruitment of endothelial cells and pericytes [6, 8]. In addition, breast carcinoma cells produce MMP9 [9–11] and TAFs enhance expression of MMP9 by tumor cells in the breast TME [12]. Tumor blood vessels exhibit significant structural and functional abnormalities that provoke tumor hypoxia and metastatic spread [13]. However, the roles of different cellular components of the TME and their interactions in tumor angiogenesis are not fully understood.

High levels of transforming growth factor-β (TGF-β) and pro-inflammatory cytokines such as tumor necrosis factor (TNF) have been reported for breast cancers [14–17]. These cytokines are upregulated by TAFs and can co-stimulate expression of MMP9 in the breast TME [12]. TGF-β signaling plays a critical role in breast carcinoma vascularization [9] as well as in normal vascular and cardiac development [18]. Mice lacking TGF-β type I receptor (*Tgfbr1/Alk5*) exhibit severe defects in the vascular development [19]. Endothelial cells (ECs) from Tgfbr1-mutant mice show enhanced proliferation, improper migratory behavior, and impaired fibronectin expression. It is still unclear how EC-intrinsic defects lead to vascular abnormalities. Several human syndromes including Marfan and Loeys-Dietz (LDS) are also associated with defects in the TGF-β pathway. However, the cell and molecular mechanisms underlying vascular abnormalities in these patients have not been defined.

Recent studies of breast carcinoma xenografts revealed that TAFs enhance tumor vasculature via a mechanism involving tumor TGF-β signaling [12]. The current study examined the effects of TAFs on the structure of tumor blood vessels and the role of tumor TGF-β signaling in this response. The study found that TAFs enhanced coverage of endothelium by pericytes, vascular mural cells supporting blood vessels. Tumors with active TGFBR1 receptor increased amounts of TAFs in the TME and enhanced tumor vasculature with improved coverage of endothelium by pericytes. In contrast, inactive TGF-β signaling reduced amounts of fibroblasts and the association of pericytes with endothelium. Accordingly, tumors expressing inactive-TGFBR1 showed signs of hemorrhages. Together, these results indicate that TGF-β signaling is important for TAF-stimulated tumor vascularization.

## Methods

### Antibodies and other reagents

Human TGF-β1 (Cat#240-B/CF) was from R&D Systems (Minneapolis, MN). Antibodies for: GAPDH (sc-25,778), p38MAPK(sc-81,621), haemaglutinin epitope (rabbit polyclonal Y-11; sc-805), SMA (sc-32,251) were from Santa Cruz Biotechnology, Inc. (Santa Cruz, CA); phospho-Smad2 (#3108), phospho-HSP27 (#2401), phospho-p38 (#9211), RELA/p65 (#8242) were from Cell Signaling Technology (Danvers, MA); α-Tubulin (#T6074), α-Catenin (#C2081) and FLAG (#F3165) were from Sigma-Aldrich (St. Louis, MO); FN1 (#610077) and Smad2 (#610842) were from BD Biosciences (San Jose, CA); Ki67 were from Abcam (#ab833). Goat anti-Rabbit IgG (H + L)-Horseradish Peroxidase (HRP) (#170–6515) and goat anti-Mouse IgG (H + L)-HRP (#170–6516) secondary antibodies were from BIO-RAD Laboratories (Hercules, CA). Retroviral constructs encoding EGFP, HA-tagged TGF-β type I receptor TGFBR1/ALK5-wild-type, TGFBR1-K232R and TGFBR1-T204D mutants are described in [20]. SMAD3 and SMAD4 constructs, FN1-lux luciferase reporter are described in [21].

### Cell culture

Human breast carcinoma cell line MDA-MB-231, rat embryonic fibroblast cell line 208F, human embryonic fibroblast cell line WI-38, human HEK-293 T and mouse mammary epithelial NMuMG cell line were obtained from American Tissue Culture Collection (ATCC) (Manassas, VA) and cultured as recommended by ATCC. MDA-MB-231 cells expressing EGFP-only, HA-tagged inactive TGFBR1/ALK5-K232R and active TGFBR1-T204D receptors were generated by retroviral transduction and are described elsewhere [9, 20]. Mouse fibroblasts from wild-type and *Smad3*-deficient mice [22] were a generous gift of Drs. Anita Roberts and Kathy Flanders (National Institute of Health).

### Adenoviral infection of cells

Adenoviruses encoding EGFP, Flag-tagged SMADs, and HA-tagged constitutively-active mutants TGFBR1/ALK5-T204D and BMPR1A/ALK3-Q233D were produced using HEK-293 T cells and stored in aliquots at − 80 °C as described in [23, 24]. Cells were incubated for 3 h with supernatant containing adenoviruses at 5–10 MOI. Medium was replenished and cells were grown for additional 24 h before further treatments.

### Immunoblot analysis

Immunoblotting was done as described in previous studies [25]. Briefly, whole-cell lysates were collected using NP40 Lysis Buffer. Where it is indicated cells were treated with 2 ng/mL TGF-β1, and inhibitors were added 1 h prior to cytokine treatment. Proteins were resolved on SDS-PAGE and the bands were visualized using ECL chemiluminescent reagent (#32209; Pierce).

### Luciferase reporter assay

NMuMG cells were transfected with the firefly luciferase (Luc) reporter FN1-Lux (1 μg) and pCMV–*Renilla reniformis* luciferase (0.025 μg) (Rl; Promega) in 6-well plates (10^6^ cells/well) using FuGENE 6 reagent and according to the manufacturer’s protocol. The next day, cells were transferred into a 48-well plate (2 × 10^4^ cells/well). The cells were treated with 2 ng/ml TGF-β1 for 24 h. Luc and Rl activities were determined using the Dual-Luciferase reporter assay system (Promega), according to the manufacturer’s protocol, in a microplate luminometer (Veritas; Promega). Firefly activity was normalized to Renilla activity and presented as relative luciferase units. All assays were performed in triplicates, and each experiment was repeated at least twice.

### Fluorescence microscopy

Cells were grown on glass coverslips (22 × 22 mm) and treated with 2 ng/ml TGF-β1 for 24 h. The cells were fixed with 4% PFA and permeabilized with 0.05% Triton X-100 and then blocked with 3% milk in PBS for 30 min at room temperature. The cells were incubated for 1 h with antibodies to fibronectin (1:400) in 1% milk/PBS followed by incubation for 30 min with Texas red–conjugated secondary antibody (1:500) at room temperature. Fluorescence images were taken with a Plan Apochromat 60×/1.40 NA oil objective lens at ambient temperature using an inverted microscope (TE2000-E; Nikon) equipped with a charge-coupled device camera (CoolSNAP HQ; Photometrics). The images were acquired using MetaVue imaging software (v7.7.3, Molecular Devices).

### Animal housing

Female SCID/CB17 mice, 6–7 weeks of age, were obtained from a colony of SCID/CB17 mice bred and maintained at the Department of Laboratory Animal Resources (DLAR) facility at the Roswell Park Cancer Institute (RPCI). Animals were kept 4–5 mice per cage in microinsulator units and provided with food and water ad libitum according to a protocol and guidelines approved by the Institute Animal Care and Use Committee (IACUC) at the Roswell Park Cancer Institute. The facility is certified by the American Association for Accreditation of Laboratory Animal Care (AAALAC) and in accordance with current regulation and standards of the US Department of Agriculture and the US Department of Health and Human Services.

### Animal studies

The tumor cell inoculation was done as described in [12]. Briefly, exponentially growing breast cancer cells (1.5 × 10^6^) in 0.1 mL sterile phosphate buffered solution (PBS) supplemented with reduced growth factor basement membrane extract were injected with a 27G needle into the left flank of 8-week old female SCID mice (six mice per group). For admixture experiments, tumor cells (1.5 × 10^6^) were mixed in a 3:1 ratio with fibroblast cells prior to injection into the same mice. Tumor diameters were measured with electronic calipers every 2–3 days. Volumes were calculated using the formula (length) × (width)^2^/2. Mice were humanely euthanized using CO_2_ asphyxiation followed by cervical dislocation and tumors were collected for histological analysis at the RPCI Pathology Core Facility.

### Immunohistochemistry

Tumors were excised and processed as described in [12]. Briefly, tumors were fixed in 10% (*v*/v) formalin or Zinc Fixative (#550523; BD Biosciences, NJ), for CD31 staining, before embedding in paraffin. H&E-stained sections were prepared and CD31 staining was done with rat anti-mouse CD31 antibody (#550274, BD Biosciences) and biotinylated secondary anti-rat antibody (BD Biosciences) as described in [9]. Microvessel density was analyzed as described in [26]. Briefly, tumor sections were scanned at × 100 magnification for areas containing the highest number of discrete CD31-positive microvessels (microvessel hot spots). Necrotic and immediately adjacent areas were excluded from counting. CD31-positive vessels were counted at × 400 magnification in 8 fields of each tumor section. Results were presented as mean number of microvessels/field (0.2 mm^2^) ± standard deviation. Rat fibroblasts were detected with an antibody to prolyl 4-hydroxylase (6-9H6) (NBP2–33342; Novus Biologicals; Littleton, CO).

The luminal size of vessels was evaluated on CD31-stained sections as described in [27]. Briefly, the areas with the largest blood vessels were identified at low magnification, and the diameters of the five largest vessels in each of five microscopic fields were measured at × 200 magnification in five tumors from each group. The results were presented as the mean number of diameters in each group ± standard deviation. Fibroblast presence was evaluated using smooth-muscle actin (SMA) antibody (A-2547, Clone 1A4, Sigma) and quantified using NIH ImageJ software. RGB-images of SMA-stained sections were recorded at × 200 magnification and imported into ImageJ software. Blue-channel images were extracted to eliminate background stain and SMA-positive staining was measured using ImageJ software. The average values of the SMA-positive areas and standard deviations were calculated relative to the total area of the tumor section (%) for 2–4 visual fields from 3 to 6 tumors per group. Ki-67 staining was done on formalin-fixed sections using rabbit polyclonal antibodies to human Ki-67 and biotinylated secondary goat anti-rabbit antibodies with the ABC reagent (Vector Labs). At least 400 tumor cells per specimen were examined in five random fields at × 600 magnification. Ki-67 labeling index was calculated as the percentage of Ki67-positive nuclei relative to the total number of cells examined.

For evaluation of the pericyte-endothelium association, Zinc-fixed tissue sections were double stained for CD31 and NG2/CSPG4 antibodies (AB5320, Chemicon, MilliporeSigma). RGB-images of the CD31/NG2 double-stained tissue sections were recorded at 400× magnification using a BX46 Olympus microscope as described above. Quantification of the pericyte-endothelial cell association was done from three visual fields in three tumors per group and using the NIH ImageJ manual counting application. The pericyte-endothelium association was calculated as the percentage of endothelial cells (CD31) associated with pericytes divided by the total number of CD31-positive vessels examined. The quantification of the pericyte presence within the tumor areas outside of blood vessels was determined using NIH Fiji ImageJ2 [28]. RGB-image files (TIFF) were imported into the ImageJ2 software and then carefully selected to exclude any blood vessels or necrotic tissue. Images underwent colour deconvolution and were split into three channels; the brown-coloured channel, corresponding to the NG2^+^ stain, was used for quantification. Quantification was done by measuring the amount of thresholded area that included only the NG2^+^ stain within the selected tumor areas. A fraction of the NG2^+^ area was calculated relative to the total area of the selected tumor sections (%). The average values of the fractions and standard deviations were done in 2 tumor areas for 3 sections from 3 tumors per group.

The presence of hemorrhage in the tumor was used as an indicator of leakage of the blood vessels. H&E-stained sections of tumors were subjected to histopathologic examination and hemorrhage was graded on a semi-quantitative scale from 0 to 4 using the following scoring chart: 0 – no hemorrhage; 1 – scant hemorrhage, one or at most two minute foci, visible at × 100 magnification; 2 – moderate hemorrhage, two to three larger foci, visible at × 40 magnification; 3 – severe hemorrhage, multifocal or confluent, visible at × 20 magnification. The average scores and standard deviations were calculated using 5 tumors per group.

### Cell death in tumor xenografts

Cell death was evaluated using Terminal deoxynucleotidyl transferase (TdT) dUTP Nick-End Labeling (TUNEL) in tumor tissues with the ApopTag Plus Peroxidase In Situ Apoptosis Detection Kit (S7101; Chemicon, MilliporeSigma). Cells were examined in 5 random fields at × 400 magnification. TUNEL-positive area within the tumor core was evaluated using NIH ImageJ software at × 40 magnification as described in [29]. Briefly, images were recorded as described above. RGB-image files (TIFF) were imported into ImageJ software and then Blue-channel images were extracted to eliminate background stain. The TUNEL-positive stained area was thresholded and measured using ImageJ software. A fraction of the TUNEL-positive area was calculated relative to the total area of the tumor (%). Tumor periphery was not included for this quantification. The average values of the TUNEL-positive area fractions and standard deviations were determined from 5 to 6 tumors/group.

### In vitro co-culture

Tumor-fibroblast co-cultures were done as described in [27]. Briefly, tumor cells were seeded at 4.5 × 10^5^ cells per well individually or as a 3:1 mixture with fibroblasts. Fibroblasts were seeded alone at 1.5 × 10^5^ cells per well. The cells were incubated for 48–72 h prior to preparation of whole-cell lysates or RNA for immunoblotting and qRT-PCR, respectfully.

### Statistical analysis

Data in each experiment was compared using the Student’s *t* test. Statistical significance was achieved when *P* < 0.05.

## Results

### Disruption of TGF-β signaling affects fibroblast-enhanced tumor growth

**Fig. 1:**
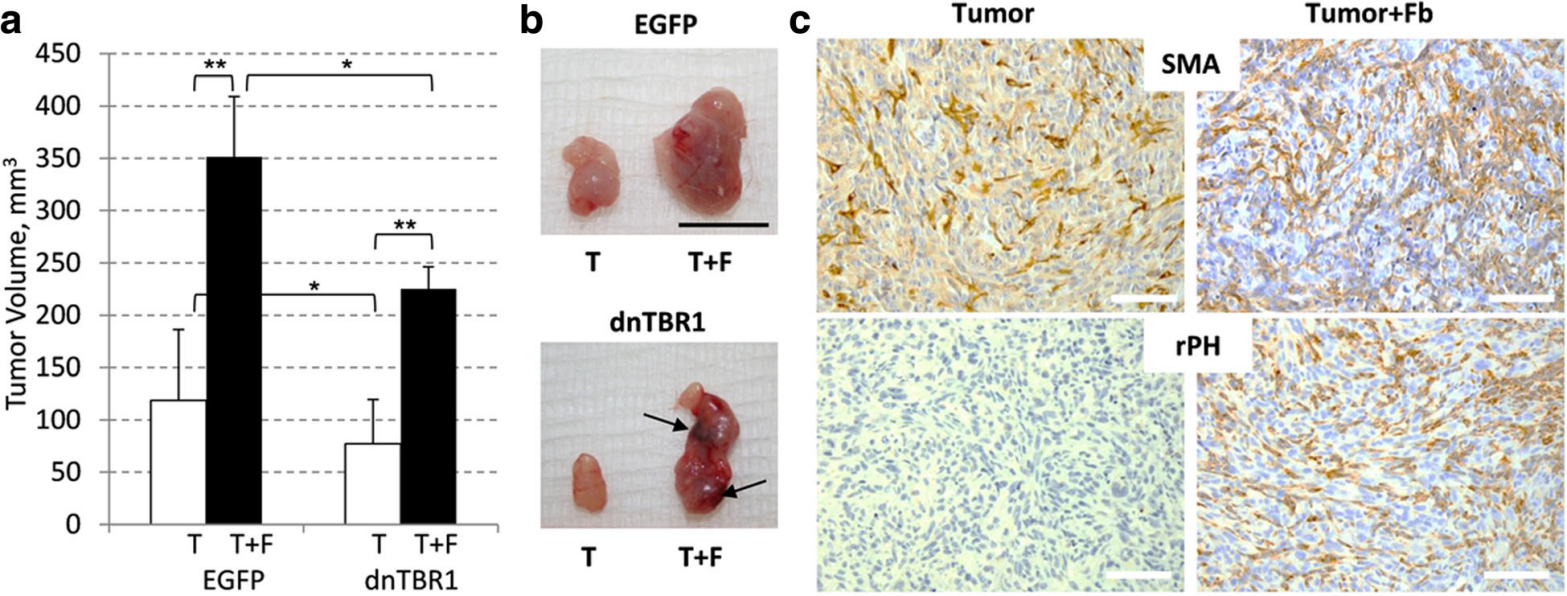
Fibroblasts enhance tumor growth. **a** Graph shows tumor volumes at the endpoint of the xenograft study. Breast carcinoma MDA-MB-231 cells, empty-vector control (EGFP) and kinase-inactive TGFBR1 (dnTBR1), were inoculated in SCID mice (6 mice/group) alone (T) or in combination with fibroblasts (T + F). **, *P* < 0.01; *, *P* < 0.05. **b** Images of tumors at the endpoint of the study. Arrows indicate sites of hemorrhages. Bar size, 10 mm. **c** Staining of fibroblasts with antibodies to smooth-muscle actin (SMA) and rPH, prolyl 4-hydroxylase, a marker of rat fibroblasts. Images were taken at × 400 magnification, bar size, 50 μm

Recent studies have revealed that a tumor-fibroblast crosstalk up-regulates TGF-β cytokine expression and signaling [12]. Further, admixture of breast cancer MDA-MB-231 cells with either human WI-38 or rat 208F fibroblasts shows enhanced tumor growth and angiogenesis [12]. The current study examined whether this tumor-fibroblast crosstalk is affected by disruption of TGF-β signaling in tumor cells. TGF-β signaling in MDA-MB-231 cells was modulated by expressing kinase-inactive (K232R) or constitutively-active (T204D) mutants of the TGFBR1/ALK5 receptor. The cell populations were biochemically characterized in a previous study [9]. Empty-vector control (EGFP) and kinase-inactive (dominant-negative, dn) TGFBR1-expressing MDA-MB-231 cells were inoculated into immune-deficient mice, alone or as admixture with non-tumor diploid 208F fibroblasts. At the end-point of the study, the tumor size of admixture xenografts was nearly three times greater compared to tumor-alone xenografts (Fig. 1a-b). Tumors with a kinase-inactive dnTGFBR1 receptor were smaller compared to controls, while fibroblasts still increased the tumor size (Fig. 1a-b). Neither fibroblasts nor dnTGFBR1 affected the proliferative Ki67 index (Additional file 1: Figure S1A). Evaluation of TUNEL staining at the tumor periphery did not show significant changes in cell death (Additional file 1: Figure S1B). However, a significant increase in TUNEL-positive areas in the tumor core was observed in dnTGFBR1-tumor admixture xenografts indicating an increase in necrosis (Additional file 1: Figure S1C). This finding is consistent with an increase in necrotic areas in dnTGFBR1 tumors found in the orthotopic model [9]. In addition, gross assessment of tumors upon necropsy revealed a large vessel supplying blood to the tumor in admixture xenografts (Additional file 2: Figure S2), suggesting enhancement of tumor vascularization.

Finally, we examined host mouse fibroblasts and supplementary rat fibroblasts in tumors. Immuno-histochemical analysis of smooth muscle actin (SMA) showed the presence of mouse fibroblasts within the tumor of both tumor-only and admixture xenografts, whereas rat fibroblast marker prolyl 4-hydroxylase, rPH, was detected only in admixture xenografts (Fig. 1c). These findings indicate that both types of xenografts accumulated mouse fibroblasts over time, while the supplementary fibroblasts were present in admixture xenografts throughout the duration of the experiment.

Thus, tumor-associated fibroblasts enhanced tumor growth and vascularization without a significant effect on the cell proliferation or death. An increase in necrotic areas of the dnTGFBR1 co-xenografts suggests a defect in blood supply to the tumor. Consistent with this idea, gross evaluation of the tumors revealed hemorrhages in the dnTGFBR1 co-xenografts, while this was not observed in empty-vector control co-xenografts (Fig. 1b and Additional file 2: Figure S2). This finding suggests that inactivation of tumor TGF-β signaling affects blood vessel organization or function, resulting in fragile blood vessels.

### Tumor TGF-β signaling affects the ability of fibroblasts to enhance tumor vascularization

**Fig. 2:**
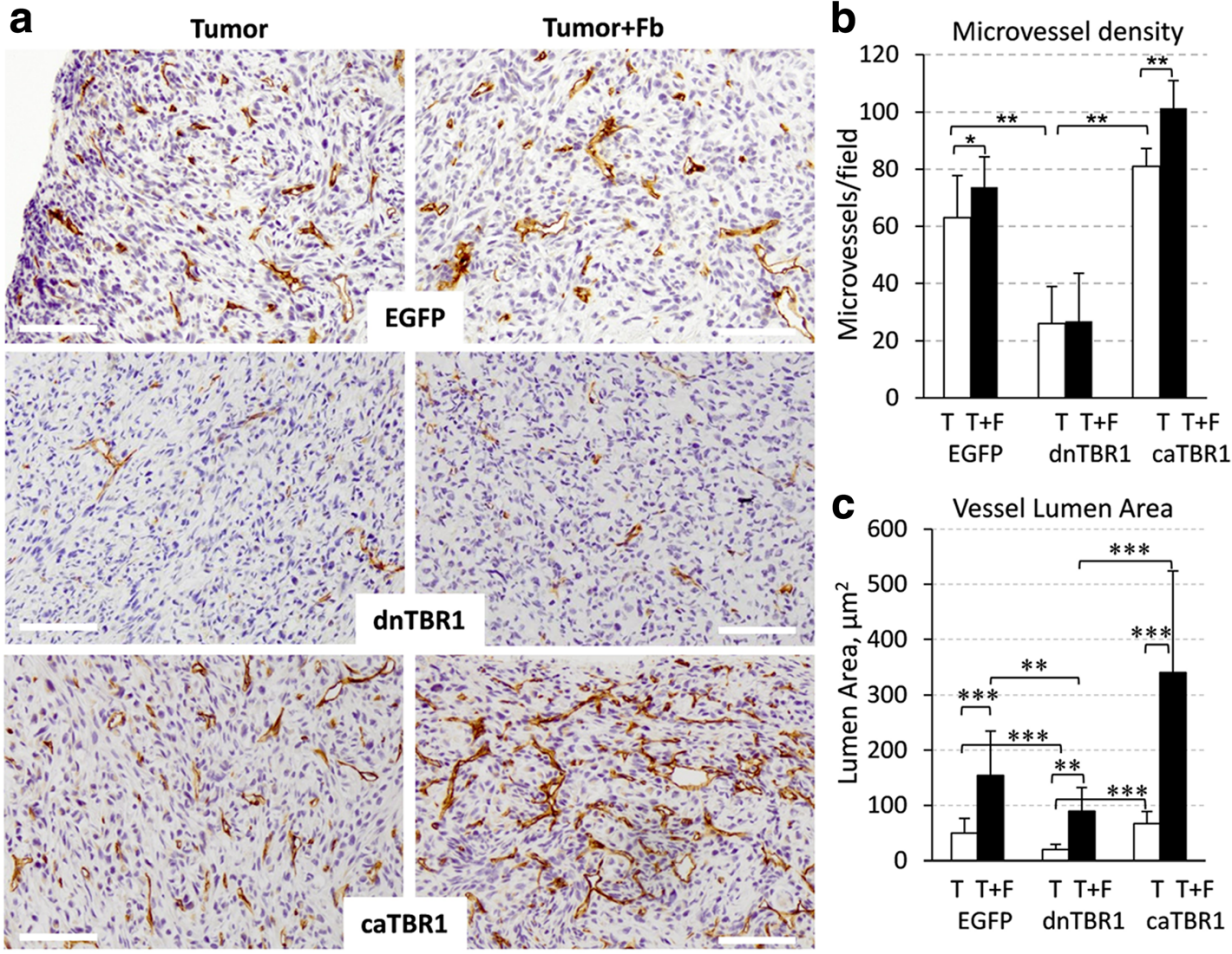
Fibroblasts enhance tumor vasculature and tumor TGF-β signaling promotes microvessel density and lumen size. **a** CD31 staining of blood vessels in tumor xenograft sections of breast carcinoma MDA-MB-231 cells, empty-vector control (EGFP), kine-inactive TGFBR1 (dnTBR1), or constitutively-active TGFBR1 (caTBR1), alone (Tumor) or in combination with fibroblasts (Tumor+Fb). **b** Quantification of the microvessel density was done using CD31 stained tumor sections in six fields for each tumor section (5 tumors/ group) and presented as a mean number per field (0.2 mm^2^). **, P < 0.01; *, P < 0.05. **c** Blood-vessel lumen area was measured at × 200 magnification in tumor sections stained for CD31, 3 tumors/group, n(T/T + F) = 60/59(EGFP); 55/56(dnTBR1); 60/54(caTBR1). **, *P* < 0.01;***, *P* < 0.001

To explore whether inactivation of TGF-β signaling influences tumor blood-vessels, tumor sections were stained for endothelial cell marker CD31/PECAM (Fig. 2a). The analysis of whole-tumor cross-sections showed that the density of microvessels was markedly reduced in dnTGFBR1-tumors (Fig. 2a) and this was statistically significant (Fig. 2b). Fibroblasts increased the vessel density and this was negated in dnTGFBR1-tumors. In comparison, tumors with constitutively-active TGFBR1 (caTGFBR1) showed increased the microvessel density compared to the control and dnTGFBR1 groups (Fig. 2a-b). Further, the lumen area of blood vessels was increased in the admixture xenografts (Fig. 2c), while in dnTGFBR1 tumors, the lumen area was reduced in both tumor-only and admixture xenografts (Fig. 2c). In contrast, the lumen area was increased in caTGFBR1-tumors (Fig. 2c). Lumen diameters were also increased by fibroblasts and active TGF-β signaling (Additional file 3: Figure S3). Together these findings indicate that fibroblasts enhance tumor vascularization, increasing the microvessel density and lumen sizes. Our results also revealed that tumor TGF-β signaling is an important player in this response.

### Inactivation of TGF-β signaling reduces amounts of tumor-associated fibroblasts

**Fig. 3:**
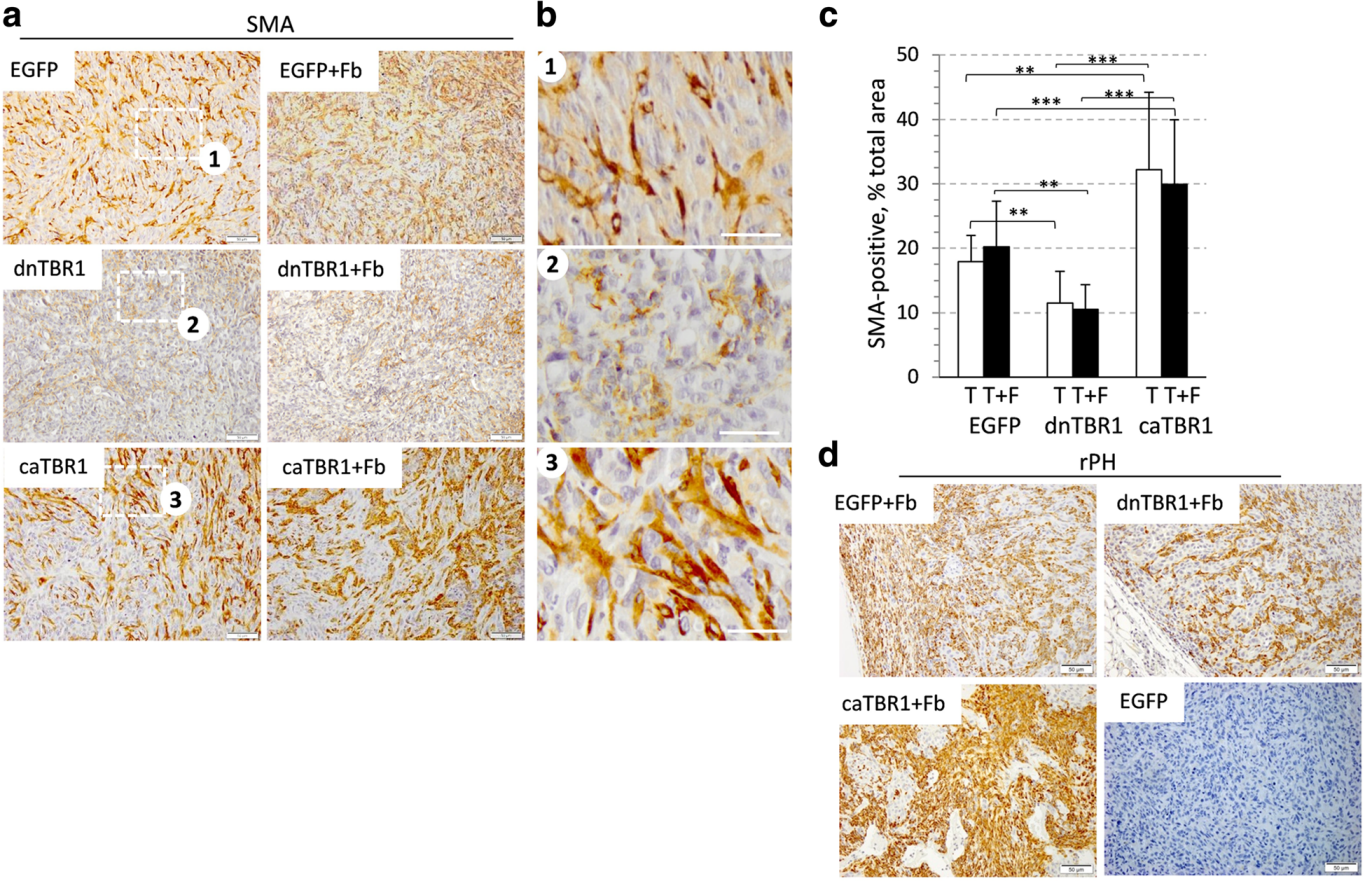
TGF-β signaling increases amounts of tumor-associated fibroblasts. **a, b, d** Detection of fibroblasts with antibodies to smooth-muscle actin SMA (**a-b**) and prolyl 4-hydroxylase rPH (**d**), a marker of rat fibroblasts, in tumor xenograft sections of carcinoma MDA-MB-231 cells: empty-vector control (EGFP), dnTGFBR1 (dnTBR1), or constitutively-active TGFBR1 (caTBR1), alone or in combination with fibroblasts. Images are taken at × 200 magnification, bar size, 50 μm. Panels in (**b**) show enlargements of marked areas in (**a**), bar size 25 μm. **c** Quantification of SMA-positive cells using images taken at × 200 magnification EGFP (6 tumors/group; field images *n* = 15, 17); dnTGFBR1 (3 tumors/group, *n* = 9, 8); caTGFBR1 (5 tumors/group, *n* = 14, 16). *, *P* < 0.05; **, *P* < 0.01; ***, *P* < 0.001

To examine whether inactivation of TGF-β signaling influences tumor-associated fibroblasts, tumor sections were stained for fibroblast markers, smooth-muscle actin SMA and rPH, a rat fibroblast-specific marker (Fig. 3). The analysis of tumor-only sections (Fig. 3a, left panels; 3B) showed that amounts of SMA-positive cells, mouse fibroblasts, were visibly reduced in the dnTGFBR1 group compared to the EGFP-control group (quantified in Fig. 3c). A reduction in SMA-positive cells was also noted in tumor-fibroblast co-xenografts of the dnTGFBR1 group (Fig. 3a, right panels). In contrast, caTGFBR1-tumors exhibited greater presence of SMA-positive cells compared to two other groups (Fig. 3a-c). In addition, we noted that fibroblasts in caTGBR1 tumors were more elongated/spread compared to EGFP-control counterparts, whereas fibroblasts of dnTGFBR1-tumors did not show this feature (Fig. 3b). The analysis of rPH staining revealed a similar trend in reduction of fibroblasts in dnTGFBR1 tumors and increased amounts and a spreading phenotype in caTGFBR1 tumors (Fig. 3d). Together, these observations indicate that tumor TGF-β signaling affects tumor-associated fibroblasts as well as their shape, i.e. active TGF-β signaling increases amounts of fibroblasts and promotes their spreading appearance while inactive TGF-β signaling reduces these responses. Changes in the fibroblast morphology may associate with TGF-β-induced expression of matrix proteins such as collagens and fibronectin.

### Fibroblasts enhance the pericyte-endothelium association and TGF-β signaling promotes this response

**Fig. 4:**
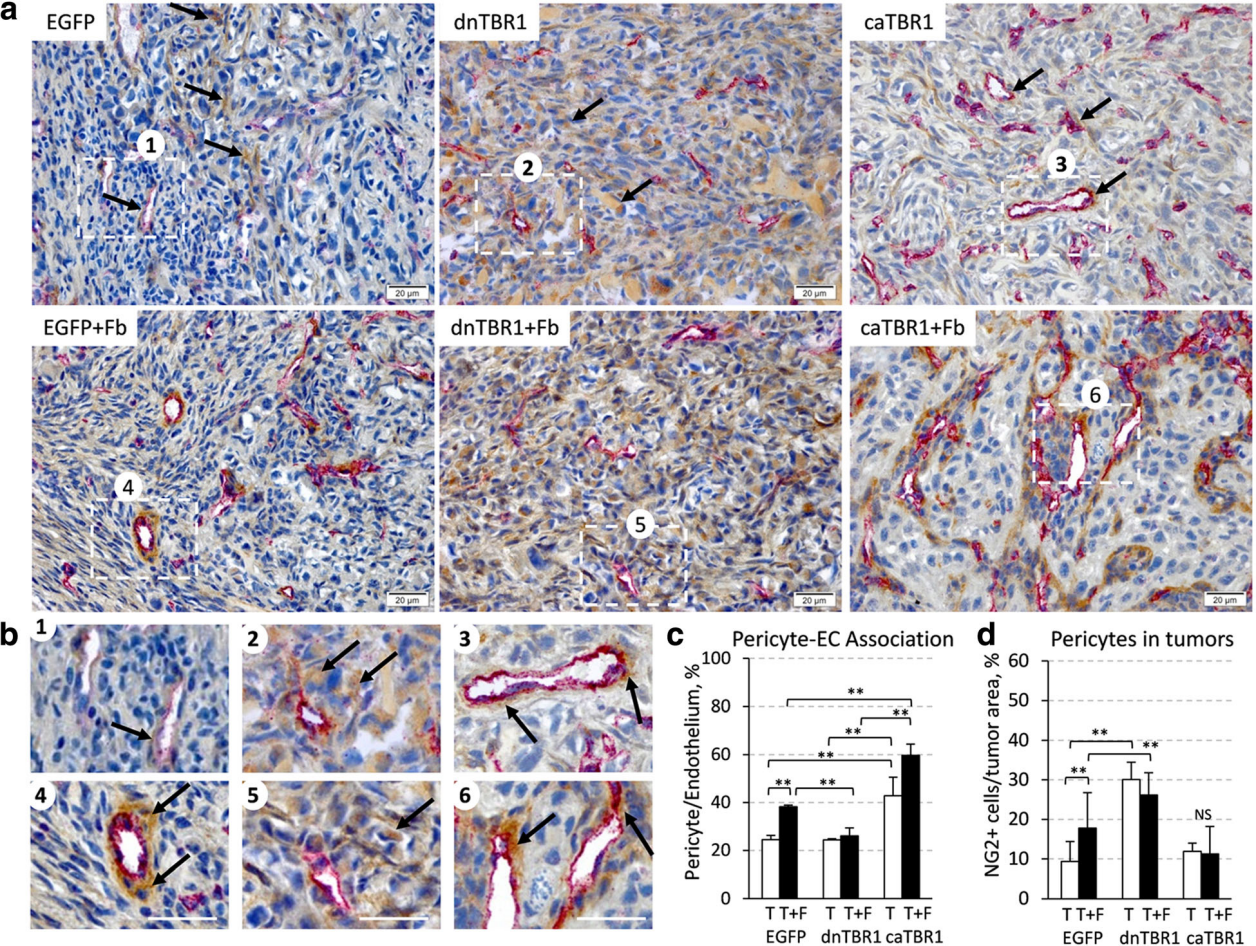
Fibroblasts and TGF-β signaling enhance coverage of endothelial cells by pericytes in tumor blood microvessels. **a** Blood microvessels in tumor sections were stained for CD31 (endothelial cells, Red) and NG2 (pericytes, Brown). Images tumor xenograft sections of carcinoma MDA-MB-231 cells, empty-vector control (EGFP), dnTGFBR1, or constitutively-active TGFBR1 (caTGFBR1), alone and in combination with fibroblasts, × 400 magnification, bar size 20 μm. **b** Enlargements of the highlighted areas, bar size 20 μm. **c** Quantification of blood vessels (CD31) associated with pericytes (NG2) in tumor tissues; 3 tumors/group, 3 sections/tumor; **, *P* < 0.01. **d** Quantification of pericytes (NG2) in tumor sections that are not associated with endothelium (CD31); 3 tumors/group, 3 sections/tumor; **, P < 0.01

Gross evaluation of dnTGFBR1 tumors showed blood vessels with abnormal curvy appearance and multiple signs of blood leakage; while caTGFBR1 tumors display these features (Additional file 2: Figure S2). To examine whether inactivation of TGF-β signaling influences the organization of tumor blood vessels, tumor sections were co-stained for CD31/PECAM, an endothelium marker, and NG2/CSPG4, a marker of pericytes, blood vessel-supporting cells (Fig. 4). In EGFP-control tumor-only xenografts, NG2-positive cells (brown) were found in the association with CD31-stained endothelium of microvessels as well as within the tumor tissue (Fig. 4a and insert 1 in b). In the admixture xenografts, NG2-positive cells (NG2^+^, pericytes) were primarily seen in a tight association with CD31-positive (CD31^+^, endothelial) cells (Fig. 4a-b, insert 4), and fibroblasts significantly enhanced the pericyte-endothelium association (Fig. 4c). In dnTGFBR1-tumors, NG2^+^ cells were present throughout the tumor tissue and around CD31^+^ cells, while their amounts appear to be increased (Fig. 4a, b-2). Addition of fibroblasts did not increase the pericyte-endothelium association (Fig. 4a-c). In contrast, caTGFBR1-tumors showed a significantly enhanced the pericyte-endothelium association (Fig. 4a-c), and fibroblasts further increased the coverage of endothelium by pericytes (Fig. 4a-caTBR1−/+Fb; 4b-3, − 6; C). Together, these findings indicate that a) fibroblasts enhance the pericyte-endothelium association; b) TGF-β signaling in tumor cells facilitates the pericyte-endothelium interaction, whereas inactivation of TGF-β signaling disrupts this response.

### Tumor TGF-β signaling stimulates expression of fibronectin via SMAD transcription factors

**Fig. 5:**
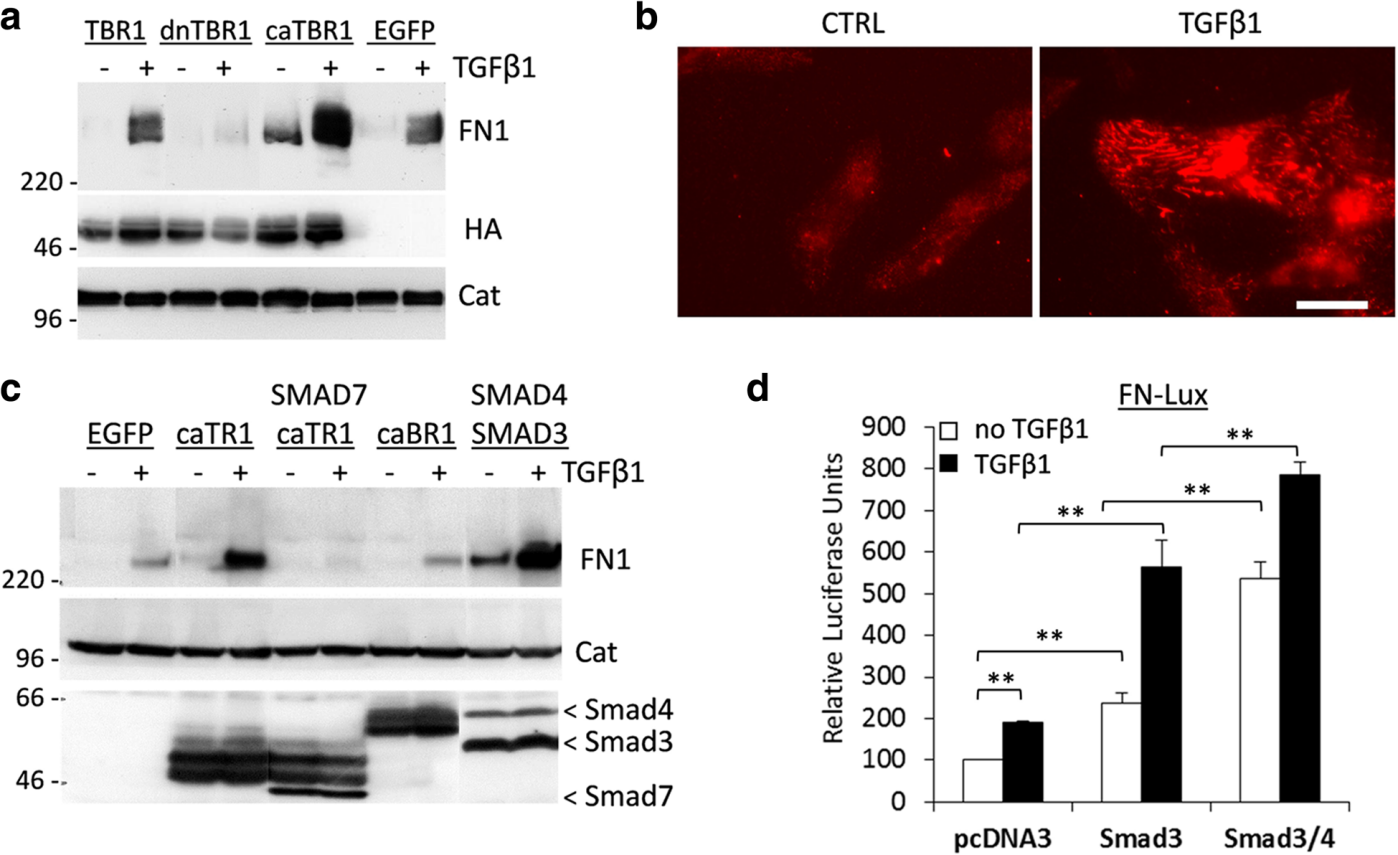
TGFβ-SMAD signaling stimulates expression of fibronectin. **a** Immunoblot analysis of fibronectin protein in MDA-MB-231 cells expressing EGFP-only (EGFP) or HA-tagged wild-type TGFBR1 (TBR1), inactive TGFBR1 (dnTBR1), or constitutively active TGFBR1 (caTBR1). α-Catenin is a loading marker. **b** Immunofluorescence images of fibronectin protein before and after 24 h-treatment with 2 ng/ml TGF-β1 of MDA-MB-231 cells, bar size 20 μm. **c** Fibronectin protein levels in MDA-MB-231 cells infected with adenoviruses encoding EGFP, caTGFBR1, caBMPR1A, caTGFBR1 and SMAD7 (a negative regulator of TGF-β signaling), or transcription factors SMAD3 and SMAD4. Expression of SMAD3, SMAD4 and SMAD7 is validated using antibody to FLAG-tag, while TGF-β and BMP receptors were detected with antibodies to HA-tag. **d** Luciferase activities of FN1-Lux reporter were measured in NMuMG cells transfected with Smad3 or Smad3/Smad4 combination. Cells were treated with 1 ng/ml TGF-β1 for 16 h. **, *P* < 0.01

**Fig. 6:**
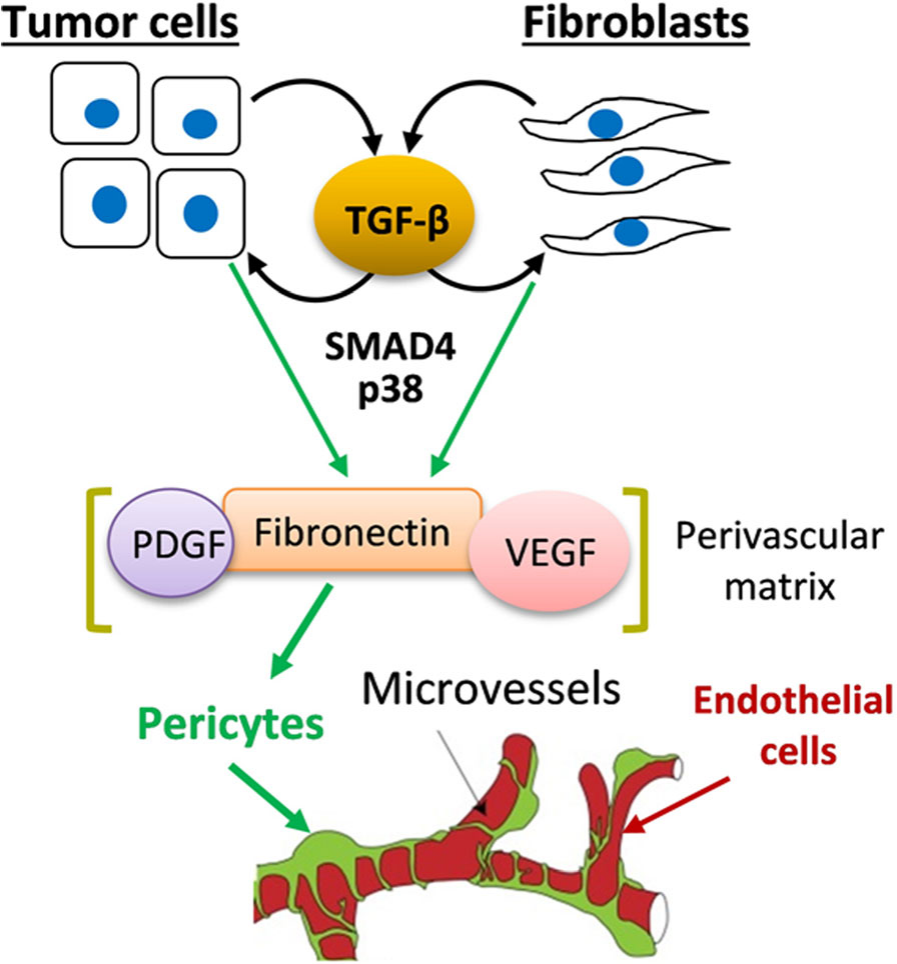
A working model of blood vessel regulation by tumor-fibroblast interactions. Tumor-fibroblast crosstalk increases levels of TGF-β cytokines which act upon tumor and fibroblast cells to stimulate expression of matrix proteins such as fibronectin. In extracellular space, fibronectin may function as a reservoir for pro-angiogenic factors PDGFs and VEGF as well as a facilitator of perivascular matrix formation affecting pericyte interactions with endothelial cells. TGFβ-SMAD signaling may also support fibroblasts via a mechanism involving fibronectin and/or other mediators

To assess how TAFs and TGF-β signaling may regulate the pericyte-endothelium association, we examined expression of fibronectin, an important component of the vascular matrix [30]. Fibronectin contributes to the vascular basement-membrane formation during endothelium-pericyte tube assembly and disruption of fibronectin fibrils impairs the pericyte-endothelium association [31]. Fibronectin is also implicated in various hemorrhagic diseases [32]. We first compared fibronectin protein levels in MDA-MB-231 expressing empty-vector (EGFP), wild-type TGFBR1, dnTGFBR1, and caTGFBR1. Immunoblot analysis revealed that TGF-β1 stimulated expression of fibronectin in EGFP-control and wild-type TGFBR1 cells whereas in dnTGFBR1 cells this response was blocked (Fig. 5a). Active caTGFBR1 increased basal and TGF-β-induced fibronectin protein levels (Fig. 5a, lane caTBR1). Expression levels of HA-tagged TGFBR1 constructs were comparable (Fig. 5a). Elevated fibronectin levels were also found in tumor-fibroblast co-cultures (Additional file 4: Figure S4), confirming a recent report [27]. Next, immunofluorescence imaging showed that TGF-β1 markedly increased deposition of fibronectin fibrils (Fig. 5b). To assess a TGF-β signaling mechanism, we examined the role of SMADs in fibronectin expression using adenoviral transduction of negative regulator SMAD7, or transcription factors SMAD3 and SMAD4. Adenoviral transduction of caTGFBR1 (caTR1) increased basal and TGF-β1-induced fibronectin levels whereas this response was blocked by co-transduction of SMAD7 (Fig. 5c). Co-transduction of SMAD3 and SMAD4 markedly increased basal and TGF-β1-induced fibronectin levels (Fig. 5c). Interestingly, transduction of active BMPR1A receptor did not have a significant impact on fibronectin expression (Fig. 5c, caBR1). To validate the role of SMAD3, we examined the response in mouse embryonic fibroblasts (MEFs) from wild-type (wt) and *Smad3*-deficient mice. Treatment with TGF-β1 induced expression of fibronectin in wt-MEFs whereas basal and TGFβ1-induced levels of fibronectin were reduced in *Smad3*-mutant MEFs (Additional file 5: Figure S5). Next, we assessed the effects of SMAD3 and SMAD4 on a luciferase activity of the FN1-lux reporter containing a fragment of the proximal promoter region of human FN1 (Fig. 5d). Transient-transcription assays showed a 2-fold induction of the luciferase-reporter activity by TGF-β1 (Fig. 5d, pcDNA3). Co-transfection of SMAD3 increased basal and TGF-β1-induced responses, while co-transfection of both SMAD3 and SMAD4 stimulated even greater the FN1-lux activity (Fig. 5d). These data show that TGFBR1-SMAD3/4 signaling up-regulates expression and deposition of fibronectin. This observation is consistent with a previous report on the role of Smad4 in the regulation of fibronectin in mammary epithelial cells [21]. Thus, the tumor-fibroblast crosstalk may promote tumor angiogenesis by activating the TGFBR1-SMAD signaling pathway, which, in turn, stimulates deposition of fibronectin fibrils promoting the endothelium-pericyte association (Fig. 6).

## Discussion

This study revealed that tumor-associated fibroblasts (TAFs) enhance tumor vascularization and tumor TGF-β signaling contributes to this response. We found that TAFs promote tumor growth (Fig. 1) and increase the lumen size of tumor blood vessels (Fig. 2). Inactivation of tumor TGF-β signaling (dnTGFBR1) reduced the microvessel density and lumen sizes (Fig. 2), decreasing tumor growth (Fig. 1). In contrast, tumors with constitutively active TGF-β signaling (caTGFBR1) exhibited greater the microvessel density and lumen sizes (Fig. 2). Inactivation of tumor TGF-β signaling decreased tumor infiltration by TAFs, while tumors with active TGF-β signaling exhibited greater presence of fibroblasts compared to control (Fig. 3). Examination of the vessel organization showed that TAFs enhanced microvessel coverage by pericytes, vascular mural cells supporting capillaries (Fig. 4). This effect was impaired in tumors with inactive TGF-β signaling, whereas active TGF-β signaling enhanced the pericyte-endothelium association (Fig. 4). Accordingly, tumors with inactive TGF-β signaling exhibited visible hemorrhages, a sign of fragile blood vessels (Fig. 1 and Additional file 2: Figure S2). Biochemical data revealed that TGFβ-SMAD signaling strongly up-regulates expression of fibronectin, which plays a prominent role in the pericyte-endothelium association [31]. Thus, our findings suggest that the tumor-fibroblast crosstalk enhances tumor vascularization by stimulating the pericyte-endothelium association via a mechanism involving the TGF-β-fibronectin axis (Fig. 6).

The current study expands our understanding of how TAFs may promote tumor vasculature and cancer progression. Previous research has implicated TAFs in recruitment of pro-angiogenic immune cells promoting tumor angiogenesis via a mechanism mediated by matrix metalloproteinase MMP9 and VEGFA [33, 34]. Recent studies have also implicated the tumor-fibroblast interactions in tumor angiogenesis by increasing expression in tumor cells of MMP9 and pro-angiogenic growth factors such as VEGFA and HB-EGF [12, 27]. Here we found that TAFs, in addition to the above mechanisms, promote maturation of blood vessels by enhancing the pericyte-endothelium association. Pericytes are embedded within the perivascular matrix and cover the walls of capillaries, providing the structural support to capillaries [35]. The capillary formation involves the recruitment of pericytes through PDGFB-PDGFRB and SDF1-CXCR4 signaling [35], while association of pericytes with endothelium is mediated by the perivascular matrix [35, 36]. Our data indicate that TAFs increased the pericyte-endothelium association but did not change tumor infiltration by pericytes. Thus, TAFs may promote the pericyte-endothelium association by regulating the perivascular matrix rather than recruitment of pericytes.

Loss-of-function and gain-of-function experiments revealed that tumor TGF-β signaling enhances tumor infiltration by fibroblasts and maturation of tumor blood vessels. Our previous work showed that the TGFBR1 activity dramatically affects the tumor cell-intrinsic metastatic potential [9]. Here, we found that TGF-β signaling augmented both host and admixed fibroblasts. However, the amount of pericytes in tumors with inactive TGF-β signaling (dnTGFBR1) was elevated compared to control and caTGFBR1 tumors. This effect may associate with hypoxic conditions observed in dnTGFBR1-tumors [9], as hypoxia stimulates recruitment of pericytes [37]. Importantly, inactivation of TGF-β signaling impaired the pericyte-endothelium association stimulated by TAFs, whereas active TGF-β enhanced the pericyte-endothelium coverage. These findings indicate that TGF-β signaling promotes maturation of blood vessels by enhancing the pericyte-endothelium interaction. Accordingly, dnTGFBR1-tumors exhibited hemorrhages and reduced growth compared to control and caTGFBR1 tumors. This observation is consistent with increased hypoxia and cell death in orthotopic xenografts of dnTGFBR1-tumor [9].

The current results suggest a possible mechanism by which TGF-β and TAFs may regulate the pericyte-endothelium association. We found that TGF-β signaling and TAFs stimulate expression of fibronectin, an extracellular matrix protein implicated in vascular development [38]. Several findings support the role of fibronectin in the pericyte-endothelium interaction. Fibronectin-deficient embryos display defects in the formation of vascular lumen and vascular network [38]. Disruption of fibronectin fibrils impairs the perivascular matrix and decreases the pericyte-endothelium association [31]. The tumor-fibroblast crosstalk increased expression of fibronectin (Additional file 4: Figure S4 and [27]). TGF-β signaling, but not BMP, induced deposition of fibronectin fibrils (Fig. 5) and myofibroblast-like phenotype associated with matrix deposition and remodeling [39]. In addition, human breast carcinomas show elevated deposits of fibronectin primarily in stromal compartments and this correlates with location of blood microvessels (Additional file 6: Figure S6). Fibronectin may promote the pericyte-endothelium association by regulating the deposition and signaling of pro-angiogenic cytokines. Fibronectin fibrils anchor VEGF-A, FGF2, and TGF-β cytokines regulating availability of active cytokines, while fibronectin-integrin interactions may facilitate cytokine release and signaling [38]. Fibronectin fibrils can also regulate vascular cell migration, differentiation, proliferation, and survival [38, 40]. Finally, fibronectin fibrils may physically facilitate the organization of the vascular basement membrane [40]. Consistent with our findings, TGF-β signaling may play a more complex role by promoting pericyte maturation as well as expression and deposition of VEGF by pericyte precursors interacting with endothelial cells [41].

Our results shed light on the function of TGF-β signaling in tumor angiogenesis. We found that TGFBR1/ALK5 signaling regulates the pericyte-endothelium association (Fig. 4). Consistent with this idea, tumors with inactive TGFBR1 exhibit hemorrhages and signs of leaky vessels (Fig. 1b and Additional file 2: Figure S2). Tumor blood spots (Additional file 2: Figure S2) are strikingly reminiscent of blood-vessel lesions (red spots or telangiectasia) typically found in patients with hereditary hemorrhagic telangiectasia (HHT) [42]. HHT is manifested by multiple red spots known as telangiectases around lips, oral mucosa, and fingertips [43]. Telangiectases consist of abnormally dilated thin-walled vessels that are prone to spontaneous and recurrent bleeding. HHT is the autosomal-dominant trait and about 90% of HHT cases are linked to genetic inactivation of the TGF-β pathway in endothelial cells [43, 44]. Recent studies have linked the pathogenesis of HHT to excessive angiogenesis and loss of capillary bed between arteries and veins [45]. In the mouse models of HHT, inactivation of ALK1, an endothelial-specific TGF-β type I receptor, or Endoglin, a TGF-β axillary receptor, results in disruption of the pericyte-endothelium communication leading to insufficient coverage of capillaries by pericytes [45]. Our data are consistent with these studies implicating TGF-β signaling in the regulation of the pericyte-endothelium association [45]. Thus, a tumor-fibroblast co-xenograft model may represent a valuable system for examining complex mechanisms underlying angiogenesis and vascular abnormalities in human diseases. In particular, it may help to define the contribution of the SMAD-fibronectin axis to the pericyte-endothelium interaction.

Our findings have broad translational implications for anti-cancer therapy targeting blood vessels. The identified role of TAFs and TGF-β signaling in the maturation of blood vessels raises a question whether targeting TAFs and/or inactivation of TGF-β would improve or worsen cancer treatment. Recent studies indicate that depletion of pericytes decreases tumor growth but markedly increases lung metastasis [29, 46, 47]. Consistent with our data, these reports show an increased capillary bleeding and a reduced tumor oxygenation. While the TGF-β pathway is a potential target in the metastatic disease, our results seed a doubt for a systemic administration of drugs inhibiting a kinase function of TGF-β receptors. In this regard, TGF-β signaling mediators such as TAK1 or p38, which also contribute to tumor angiogenesis and cancer progression [12, 20, 27, 48], may provide a better alternative strategy.

In summary, our study uncovered a novel mechanism by which TAFs may regulate tumor angiogenesis (Fig. 6). The identified tumor-fibroblast crosstalk upregulates TGF-β signaling that, in turn, increases production of fibronectin and other matrix proteins. TGF-β-stimulated matrix proteins enhance formation of the perivascular matrix, facilitating the pericyte-endothelium association.

## Conclusions

The current study indicates that tumor-fibroblast crosstalk enhances tumor vascularization by stimulating the pericyte-endothelium association via a mechanism involving TGF-β signaling. The tumor-fibroblast crosstalk upregulates TGF-β signaling that, in turn, facilitates the pericyte-endothelium association by increasing exprssion of matrix proteins. The tumor-fibroblast model may represent a useful system for dissecting the complex mechanisms governing multicellular interactions during angiogenesis.

## Additional files

Additional file 1: **Figure S1.** (**A**) Proliferative Ki67 index shows percentage of Ki67-positive cells measured from three fields in three tumors/group at × 600 magnification. Bars show the mean number of positive cells per field, NS - no statistical significance. (**B**) TUNEL (terminal deoxynucleotidyl transferase (TdT) dUTP Nick-End Labeling) staining of tumor xenografts. Images were taken at 200× magnification and TUNEL-positive cells were counted from the periphery in three fields in three tumors/group; total cell numbers/group *n* = 2796; 2962; 2923; 2528. Bars show the mean number of positive cells per field, NS - no statistical significance. (**C**) TUNEL-positive areas in the tumor core relative to total area of the tumor. Images were taken at × 40 magnification, 6 tumors in EGFP and 5 tumors in dnTGFBR1 groups; *, *P* < 0.05; **, *P* < 0.01;***, *P* < 0.001. (TIF 1356 kb)

Additional file 2: **Figure S2.** (**A**) Gross appearance of tumors: Images were taken at the time of necropsy in accordance with rules and recommendations of IACUC. Right images show enlargements of tumor-alone or tumor-fibroblast xenografts. Arrows show curly blood vessels; two stars show hemorrhages; a star shows blood leakages. (**B**) Semi-quantitative analysis of hemorrhage, an indicator of leaky blood vessels, was performed using H&E stained sections of the tumor-fibroblast co-xenografts, 5 tumors/group. **, *P* < 0.01. (TIF 4945 kb)

Additional file 3: **Figure S3.** Blood-vessel lumen diameters were measured at × 200 magnification in tumor sections stained for CD31, 5 tumors/group, n (T/T + F) = 235/202(EGFP); 137/202(dnTBR1); 120/154(caTBR1). **, *P* < 0.01;***, *P* < 0.001. (TIF 314 kb)

Additional file 4: **Figure S4.** Immunoblotting of fibronectin and GAPDH in whole-cell lysates of MDA-MB-231 (T) cells and WI-38 (F) and their co-cultures (T + F, 3:1 ratio). Cells were incubated for 48 h. (TIF 295 kb)

Additional file 5: **Figure S5.** Immunoblotting of fibronectin and alpha-catenin in whole-cell lysates from wild-type and Smad3-deficient MEFs treated with 2 ng/ml TGF-β1 for 24 h. (TIF 262 kb)

Additional file 6: **Figure S6.** Detection of Fibronectin and endothelial markers in normal breast and carcinoma tissues: (**A-B**) Immunostaining of fibronectin FN1 in normal breast (top) and breast carcinoma tissues (bottom), bar size (**A**) 200 μm and (**B**) 50 μm. (**C-D**) Immunostaining of breast carcinoma tissues for endothelial markers CD31 and CD105, bar size 200 μm. Panels in (**D**) show enlargements of highlighted areas, bar size 50 μm. The images were obtained from the Human Protein Atlas (www.proteinatlas.org), Uhlen M, Zhang C, Lee S, Sjöstedt E, Fagerberg L, Bidkhori G, et al... A pathology atlas of the human cancer transcriptome. Science. 2017;357(6352) [49]. (TIF 4246 kb)

## Abbreviations

FN1: fibronectin
TAFs: Tumor-associated fibroblasts
TAK1: TGF-beta activated kinase 1
TGF-β: transforming growth factor beta
VEGFA: vascular endothelial growth factor A
