# Supplementary figures and images for: TGF-β signaling promotes tumor vasculature by enhancing the pericyte-endothelium association
Source: BMC Cancer. 2018 Jun 19;18:670. doi: 10.1186/s12885-018-4587-z (PMC6008941; doi:10.1186/s12885-018-4587-z)

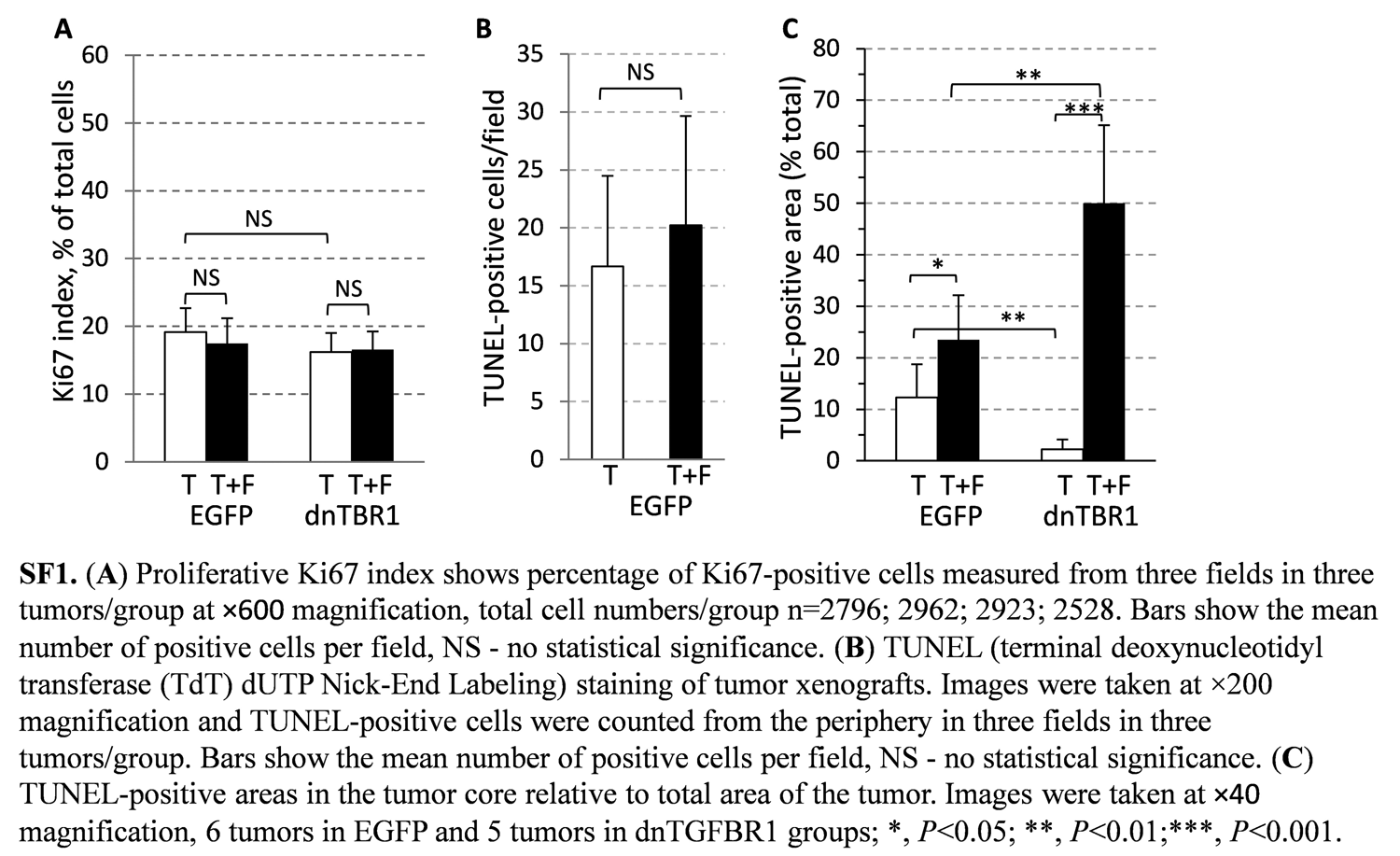

Supplement: Supplementary file 1 — Figure S1. (A) Proliferative Ki67 index shows percentage of Ki67-positive cells measured from three fields in three tumors/group at × 600 magnification. Bars show the mean number of positive cells per field, NS - no statistical significance. (B) TUNEL (terminal deoxynucleotidyl transferase (TdT) dUTP Nick-End Labeling) staining of tumor xenografts. Images were taken at 200× magnification and TUNEL-positive cells were counted from the periphery in three fields in three tumors/group; total cell numbers/group n = 2796; 2962; 2923; 2528. Bars show the mean number of positive cells per field, NS - no statistical significance. (C) TUNEL-positive areas in the tumor core relative to total area of the tumor. Images were taken at × 40 magnification, 6 tumors in EGFP and 5 tumors in dnTGFBR1 groups; *, P < 0.05; **, P < 0.01;***, P < 0.001. (TIF 1356 kb) [file 12885_2018_4587_MOESM1_ESM.tif]

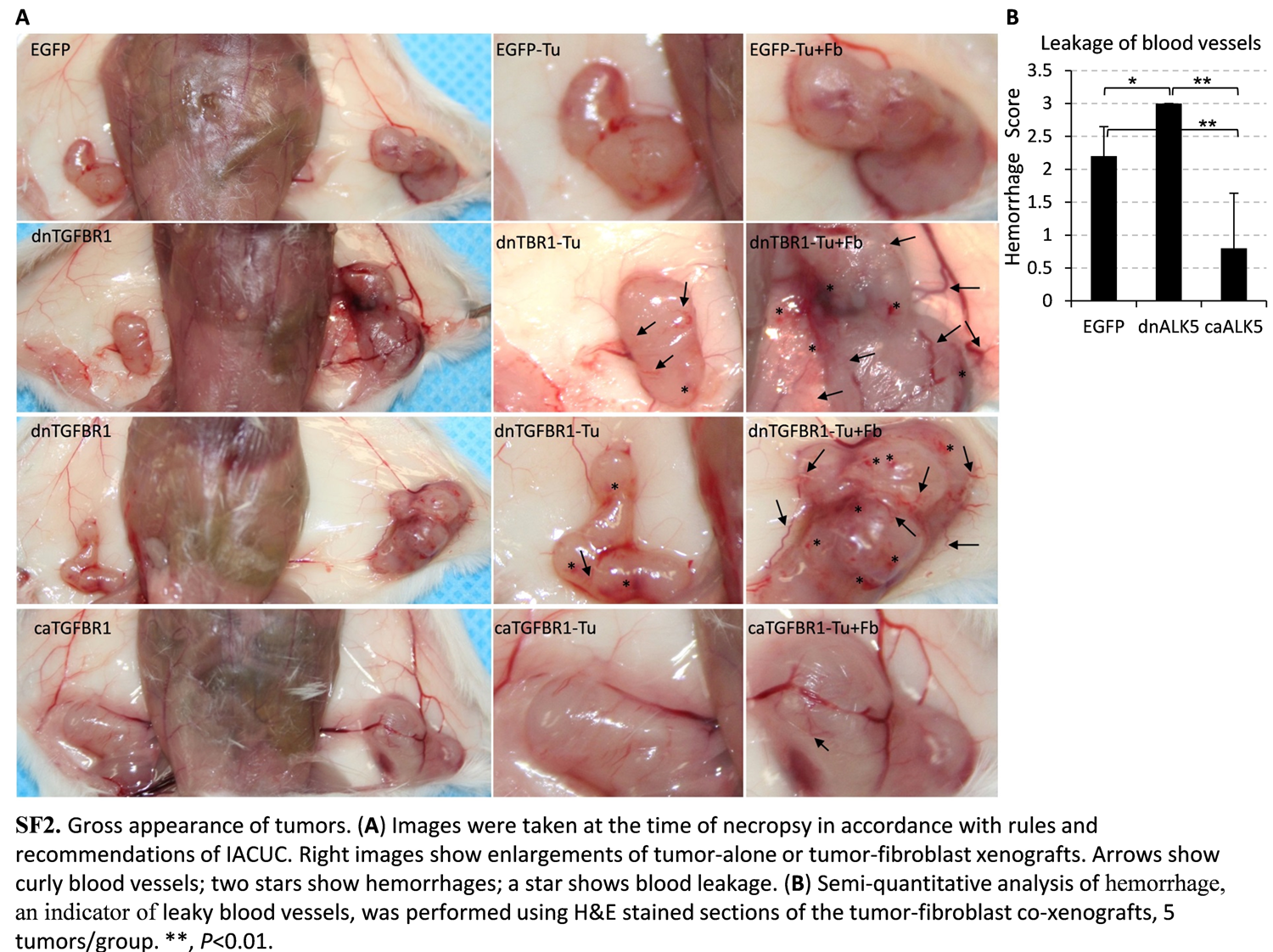

Supplement: Supplementary file 2 — Figure S2. (A) Gross appearance of tumors: Images were taken at the time of necropsy in accordance with rules and recommendations of IACUC. Right images show enlargements of tumor-alone or tumor-fibroblast xenografts. Arrows show curly blood vessels; two stars show hemorrhages; a star shows blood leakages. (B) Semi-quantitative analysis of hemorrhage, an indicator of leaky blood vessels, was performed using H&E stained sections of the tumor-fibroblast co-xenografts, 5 tumors/group. **, P < 0.01. (TIF 4945 kb) [file 12885_2018_4587_MOESM2_ESM.tif]

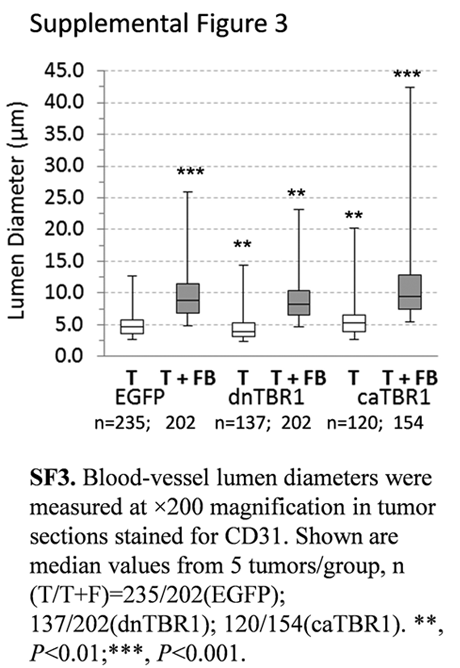

Supplement: Supplementary file 3 — Figure S3. Blood-vessel lumen diameters were measured at × 200 magnification in tumor sections stained for CD31, 5 tumors/group, n (T/T + F) = 235/202(EGFP); 137/202(dnTBR1); 120/154(caTBR1). **, P < 0.01;***, P < 0.001. (TIF 314 kb) [file 12885_2018_4587_MOESM3_ESM.tif]

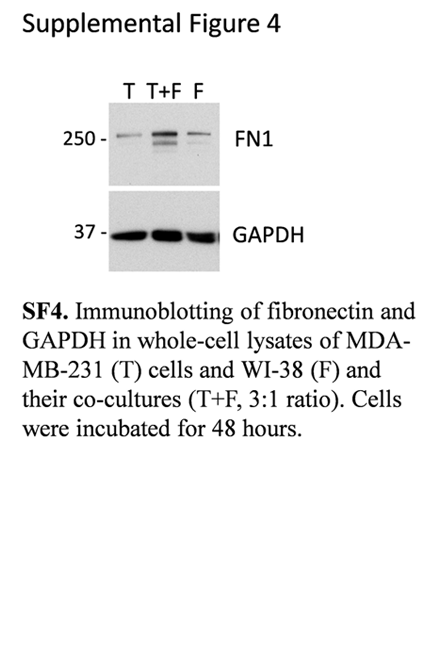

Supplement: Supplementary file 4 — Figure S4. Immunoblotting of fibronectin and GAPDH in whole-cell lysates of MDA-MB-231 (T) cells and WI-38 (F) and their co-cultures (T + F, 3:1 ratio). Cells were incubated for 48 h. (TIF 295 kb) [file 12885_2018_4587_MOESM4_ESM.tif]

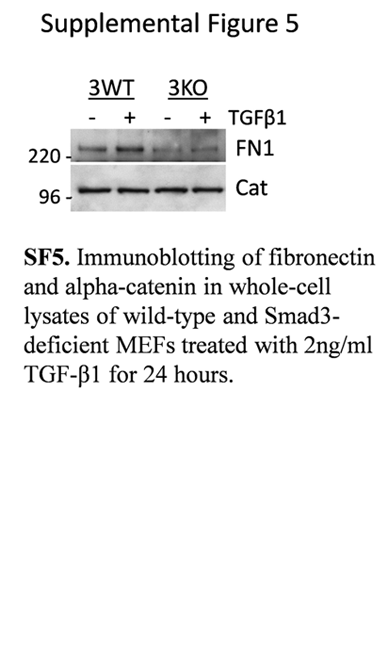

Supplement: Supplementary file 5 — Figure S5. Immunoblotting of fibronectin and alpha-catenin in whole-cell lysates from wild-type and Smad3-deficient MEFs treated with 2 ng/ml TGF-β1 for 24 h. (TIF 262 kb) [file 12885_2018_4587_MOESM5_ESM.tif]

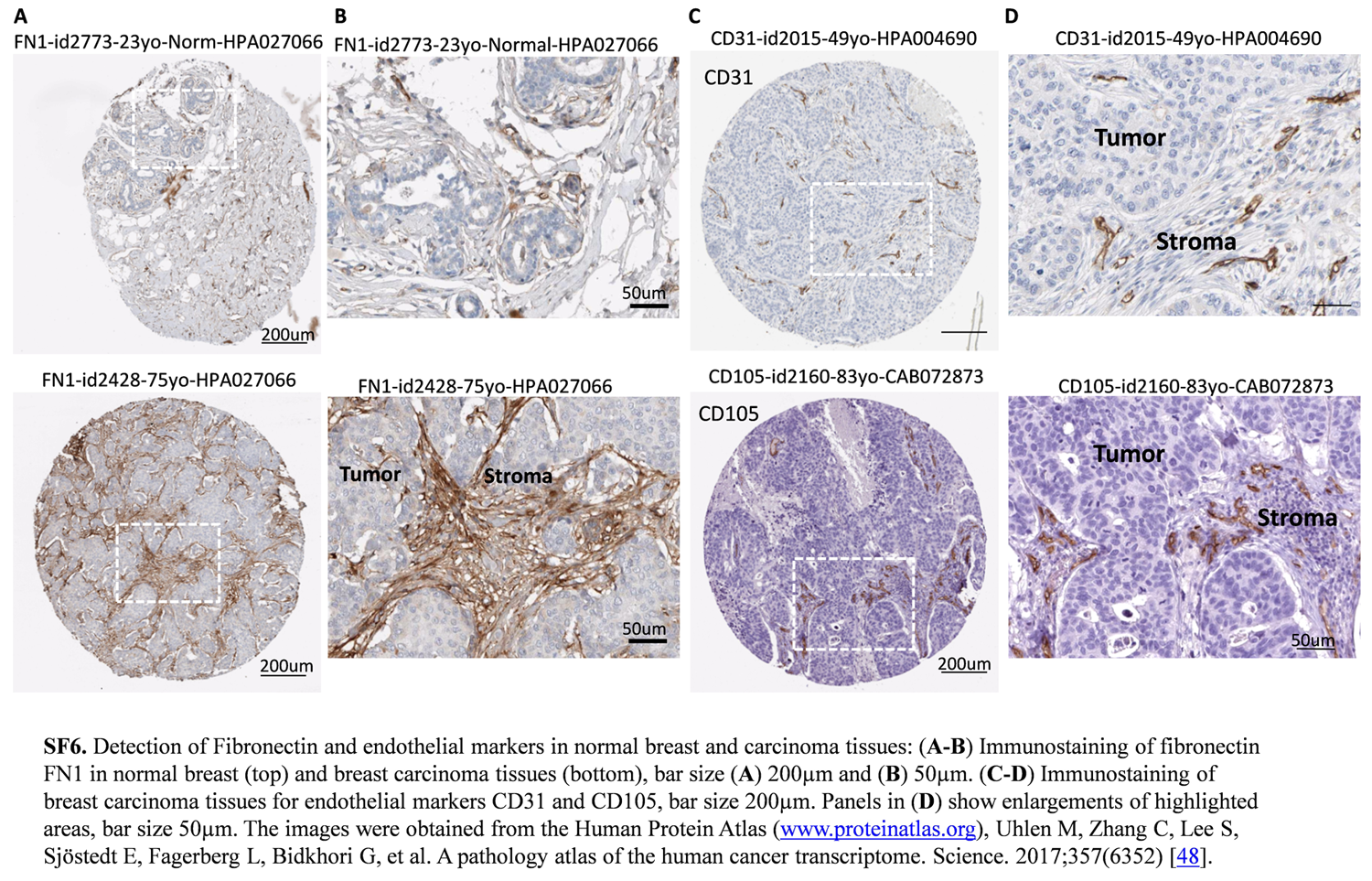

Supplement: Supplementary file 6 — Figure S6. Detection of Fibronectin and endothelial markers in normal breast and carcinoma tissues: (A-B) Immunostaining of fibronectin FN1 in normal breast (top) and breast carcinoma tissues (bottom), bar size (A) 200 μm and (B) 50 μm. (C-D) Immunostaining of breast carcinoma tissues for endothelial markers CD31 and CD105, bar size 200 μm. Panels in (D) show enlargements of highlighted areas, bar size 50 μm. The images were obtained from the Human Protein Atlas (www.proteinatlas.org), Uhlen M, Zhang C, Lee S, Sjöstedt E, Fagerberg L, Bidkhori G, et al... A pathology atlas of the human cancer transcriptome. Science. 2017;357(6352) [49]. (TIF 4246 kb) [file 12885_2018_4587_MOESM6_ESM.tif]
